# Seismic Beacon—A New Instrument for Detection of Changes in Rock Massif

**DOI:** 10.3390/s24010234

**Published:** 2023-12-31

**Authors:** Renata Lukešová, Jiří Málek

**Affiliations:** Institute of Rock Structure and Mechanics of The Czech Academy of Sciences, 182 09 Prague, Czech Republic; lukesova@irsm.cas.cz

**Keywords:** seismic beacon, nonlinear phenomena, harmonic seismic wave

## Abstract

The seismic beacon is a new instrument that allows for the measurement of changes in a rock massif with high sensitivity. It is based on effects, which affect the propagation of harmonic seismic waves generated continuously with stable and precise frequency and amplitude. These seismic waves are registered by a system of seismic stations. The amplitude of the seismic signal is very small, and it is normally hidden in a seismic noise. Special techniques are applied to increase the signal-to-noise ratio. In 2020, the first prototype of the seismic beacon was constructed in a laboratory, and field tests were performed in 2022 and 2023. During the tests, the changes in spectral amplitude and phase of seismic waves were detected, which is interpreted as the changes in material properties. These measurements testified the basic functionality of the device. The seismic beacon has been developed primarily for the detection of critical stress before an earthquake, which is manifested by non-linear effects such as higher harmonics generation. In addition, it could be used, for example, in the detection of magma movements, groundwater level changes, changes in hydrocarbon saturation in rocks during the extraction of oil and natural gas, or the penetration of gases and liquids into the earth’s crust.

## 1. Introduction

The seismic beacon was designed to measure changes in a rock massif, especially before earthquakes. During processes in earthquake sources, the strength limit of the rock is exceeded, which leads to brittle failure, rupture movement, and the emission of seismic waves. These processes are nonlinear in principle, and they can be described only very roughly with the help of the classical elastic theory. Nonlinear behavior can be observed even in the period before earthquakes, when the stress approaches the strength limit, e.g., [1,2]. This phenomenon could be the basis for earthquake forecasts in the future.

When Hook’s law (the linearity between stress and strain) is violated, there are several deviations from the elastic model of seismic wave propagation. Such nonlinear phenomena include especially generating of higher frequency harmonics, e.g., [3,4,5,6,7]. Another effect during the transition from the normal to the critical state (when the fault is near its rupture) is a slight change in the velocity and attenuation of seismic waves [8,9,10]. All these effects can be detected during the long-term operation of the seismic beacon.

It is very difficult to observe nonlinear effects in seismic waves that are generated by earthquakes, volcanic eruptions, or anthropogenic sources such as chemical explosions. These sources usually generate a continuous and relatively wide spectrum of frequencies, so the higher harmonics generated by nonlinear effects can be hidden by frequencies that are generated directly in the source. Changes in the velocity are also relatively difficult to determine, as there is a strong trade-off between the earthquake’s locations and the velocity model.

Various artificial seismic sources were developed during the last decades [11]. Besides chemical explosions, the most common devices are truck-mounted vibrators that are capable of generating vibrations with changing frequencies, so-called sweeps. However, these devices are not convenient to produce precisely repeatable signals (the contact between vibrator and soil changes with time), and the length of time intervals is limited. So, they are not very useful in measurements of small changes in the rock massif. Another possibility is to use air guns in the water. In this case, the repeatability of signals is quite good, but it produces a wide range of frequencies with insufficient spectral amplitude.

In this paper, we present for the first time the invention of a system called the seismic beacon (Czech patent PV 2020-642, international application No. PCT/CZ2021/050140). It consists of a transmitter of stable harmonic seismic waves, seismic stations situated in a special configuration, and processing software. The basic principles and functionality were verified in 2020 by construction of the laboratory model of the seismic beacon on a scale of 1:20. The next step was the construction of the first seismic beacon in the field in August 2022 and a field demonstration of this functional sample at a scale of approximately 1:3. The prototype was then improved and the next field experiment was performed in June 2023. These prototypes and performed tests are described in the present paper.

A similar methodology is used with the accurately controlled routinely operated signal system (ACROSS), described in, e.g., [12,13,14,15,16,17]. The vibrator in the ACROSS system generates a sinusoidal signal with a centrifugal force of a rotating eccentric mass. The main difference is that the seismic beacon generates continual harmonic monochromatic waves, which enables the detection of seismic waves at long distances with very low energy of generated seismic waves.

## 2. Materials and Methods

The harmonic seismic wave generator is realized in the form of a rotor, which comprises two rolling elements, moving along a circular path in a closed underground chamber. The bodies are fixed to the vertical rotor shaft and they are symmetrical. Bodies can roll in a path as the rotor moves. Both the rotor and the track are located in a closed underground chamber, for example, in a closed circular well excavated in the hard and well-elastic rock or in a concrete bunker built in the hard rock.

**Figure 1:**
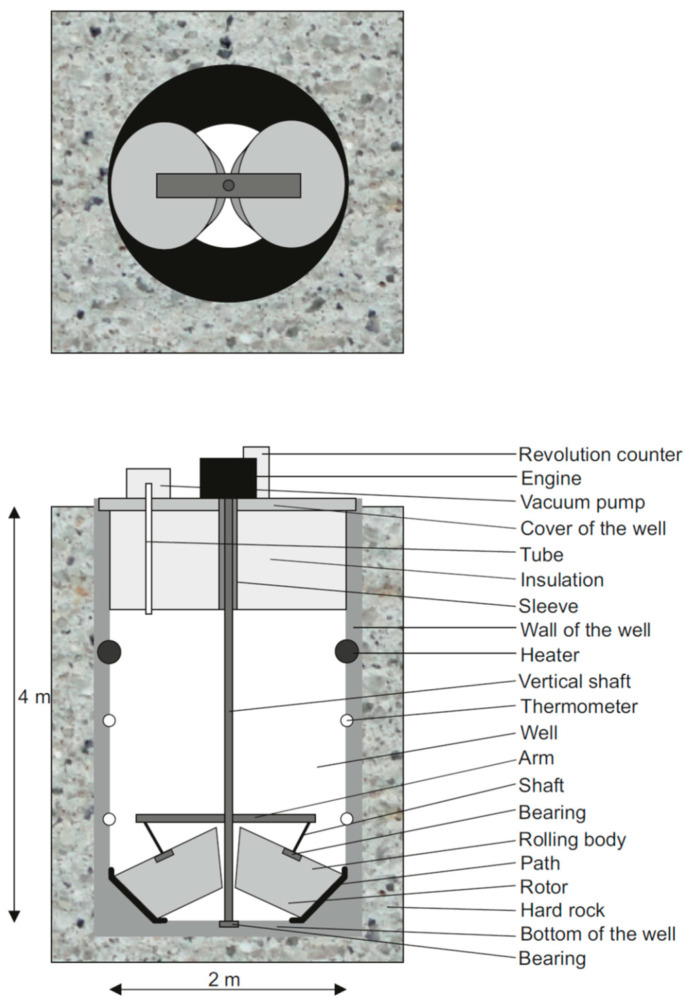
Diagram of a harmonic seismic wave generator. The upper figure shows a “cross” section—a top view of an open well. The lower figure displays side sections of the well. A rotor with two rolling elements (light grey frustums) moves around the vertical axis along a path at the bottom of the well (black thick line). The rotation is driven by a motor (black rectangle). The well is evacuated by a compressor (grey rectangle) in the top of the well and the temperature is held constant using a heater (black dots) and thermometers (white dots).

The rotor is placed in a circular well symmetrically around the axis of rotation, the path is connected to the bottom of the well. The rolling elements excite forces or force dipole on the track, resulting from the gravity and the centrifugal force. The movement of the bodies along the circular path causes a small elastic deformation of the path and, as a result, also the walls and the bottom of the well, and consequently the surrounding rocks, and thus seismic waves are generated. Both P-waves and S-waves are radiated. The maximums of P-waves are radiated in the direction of the acting forces. The diagram of the harmonic wave generator is shown in Figure 1. The diameter and depth of the well are in the range of units of meters, for example, the diameter is about 1 m to 3 m and the depth is 3 m to 5 m deep, preferably about 2 m and 4 m, respectively. The weight of each of the rolling bodies is between 500 kg to 4000 kg. Alternative designs with other shapes of rotating bodies are also possible. They are described in the patent documentation.

The receiver consists of a system of seismic stations, which are located on the surface or in boreholes around the generator. Their distance from the transmitter depends on the depth at which the changes in rock massif are expected. The transmitter-receiver geometry of the measurement must ensure that a significant part of seismic energy propagates through the volume where changes are expected. The ray path depends on the seismic velocity distribution. In most geological settings, the epicentral distance has to be at least two times bigger than the depth of the investigated section.

The processing of measurement records (seismograms) consists of monitoring the time changes of seismograms at individual seismic stations (seismometers) and also comparing the recorded seismograms between individual seismic stations. The characteristics of the seismic stations must guarantee the correct registration of the waves generated by the transmitter. The stations must be sufficiently sensitive to measure with at least the same sensitivity as natural seismic noise at the frequency considered. The sensors of the seismometer must have an appropriate frequency response (the frequency range of the sensors must cover both the basic transmitted frequency and several times higher frequencies for the detection of higher harmonic frequencies). It is preferential to use three-component stations (which register a vertical translational component and two horizontal components) or six-component stations (which, in addition to three translational components, also register 3 rotating components, see, e.g., [18,19]). The sampling rate should be at least 100 Hz. One seismic station is placed close to the generator and serves as the reference station. The processing method focuses on finding three phenomena in the propagation of seismic waves, which indicate the achievement of a critical state of tension or other changes (e.g., water saturation) in the monitored area:

- Time changes in the velocity of propagation of seismic waves;
- Time changes in seismic wave attenuation;
- Formation of higher harmonic frequencies.

When processing seismograms, it must be considered that the registered field of seismic waves does not correspond to only one wave, but it is the result of the interference of many seismic waves; body waves, surface waves as well as reflections and conversions that occur on the free surface and at geological interfaces. If a linear description of the seismic wave propagation were valid, then all these waves would have the same frequency as radiated wave. Furthermore, if the seismic wave propagation velocities and attenuation in the rock mass were independent of time, then the amplitude of the waves and the phase shift of the waves relative to the transmitter would be independent of time. The processing method, therefore, focuses on calculating whether these conditions are met or whether time dependence of velocity and attenuation are observed.

The amplitudes of the seismic waves emitted by the transmitter are weak. Therefore, there is a risk that the searched phenomena will be covered by seismic noise. Hence, the transmitted frequency should be set to the value with low seismic noise. The basic method for data interpretation is a comparison of complex Fourier spectra from seismograms registered at two stations at different epicentral distances. The ratio of the two complex spectra depends only on the geological structure, not the characteristics of the source. Moreover, the source parameters do not change in time. So, we have a double reason to interpret the time changes of complex spectra ratio as the time changes of properties of geological structure. We use very long-time windows of seismograms, typically one day. The longer the time window, the bigger the signal-to-noise ratio can be reached and the signal can be detected at more distant stations. The other methods to suppress the noise use seismic arrays as receivers [20] or denoising filters, e.g., GAS method [21].

## 3. Results

### 3.1. Laboratory Model of Seismic Beacon

**Figure 2:**
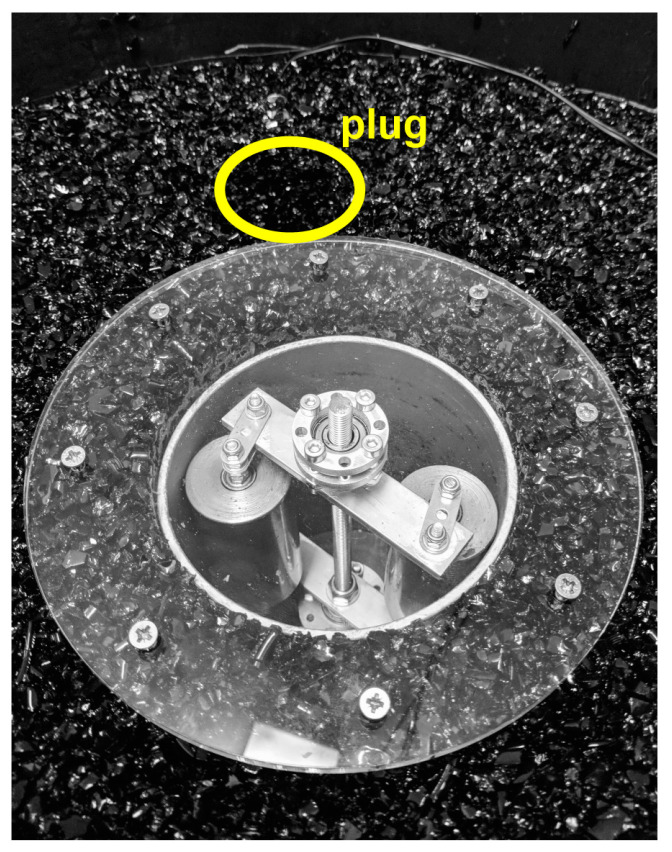
Photograph of a laboratory model of a seismic beacon. The transmitter model is on a 1:20 scale. An arrangement with cylindrical bodies and a perpendicular path along which they move is used. The yellow ellipse highlights the location of the removable plug that is used to modify conditions of the medium, where seismic waves propagate.

To verify the seismic beacon functionality and for demonstration purposes, a functional model of the seismic beacon was constructed (see Figure 2). The transmitter-generator was placed on a rubber substrate inside a round container. A seismometer, represented by an SM-6 geophone with a natural frequency of 4.5 Hz, was used as a receiver. The rolling elements of the generator were stainless steel cylinders. The movement of the rotor at 280.5 RPM (revolution per minute) produced deformations of the path, which led to the formation of seismic waves with a constant frequency of 9.35 Hz and amplitude at the location of the seismometer of approximately 4 mm/s. The model enables the production of seismic waves under different conditions, which are modified by a plug, that can be filled with various materials.

The two sensors are placed near the surface at the edge of the container. They register seismic vibrations in the parallel horizontal axis. Between the generator and one of the sensors is the plug, while the second one serves as the reference sensor. The generated seismic waves propagate through the medium and reflect from the sides and bottom of the container. The seismograms registered by geophones are therefore sum of the direct wave and multiply reflected waves.

The main difference between these laboratory model from reality is that in the real medium, the reflections from the sides does not exist. On the other hand, there are a lot of reflections from below in a real medium, so the interference of the waves also exists, and in this sense, the laboratory model fits the real situation.

Compared to standard seismic data processing, in the case of seismic beacon data, it is necessary to work with high sampling (250 Hz) and long-time series (millions of samples), which puts increased demands on calculations, memory, and computer performance. The seismic beacon signal spectrum had to be calculated from several million samples for good resolution. The methodology is based on the processing of long time series of continuous seismic noise, e.g., [22,23,24,25,26] or from groundwater level variations [27].

**Figure 3:**
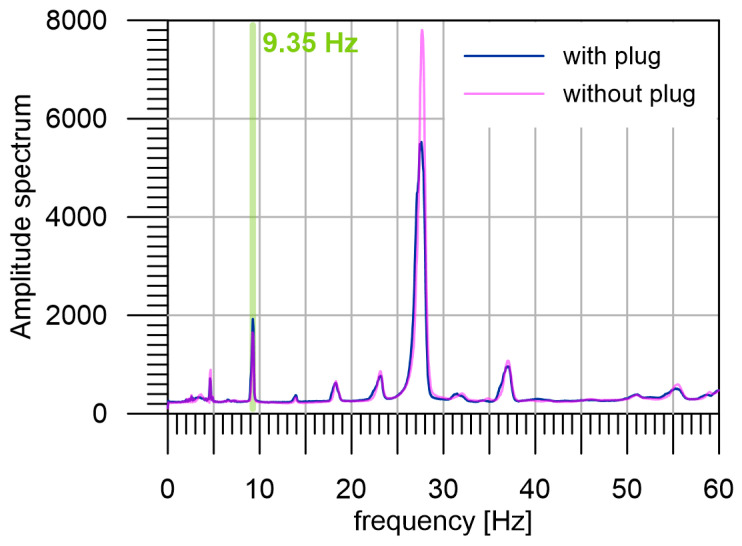
The result of a laboratory experiment with a seismic beacon model. The average amplitude spectra of the two 24 h seismograms with two various models are shown. The basic frequency of 9.35 Hz is visible and highlighted with the light green line. As a result of nonlinear effects, higher harmonics are produced at 18.70 Hz, 28.05 Hz, 37.40 Hz, and 56.10 Hz. Furthermore, the frequency 23.2 Hz, which corresponds to the natural oscillations of the model, and the frequency 4.68 Hz, which corresponds to half the fundamental frequency, are visible. The purple line represents the spectrum of the signal measured with a plug, the blue line shows the spectrum of the signal measured with plug absence.

The average amplitude spectrum of two seismograms, measured under two different model conditions is shown in Figure 3. Spectra are calculated from the 24 h measurement sections with a plug (purple line) and without a plug (blue line) on a functional model. In addition to the fundamental frequency of 9.35 Hz, we can see four higher harmonic frequencies on the spectra. The highest peak of the amplitude spectrum belongs not to the fundamental frequency (9.35 Hz) but to its third harmonic frequency (28.05 Hz). The signal measured on the model without a plug (blue line) generates an amplitude spectrum with lower amplitudes of harmonics. The ratio of amplitudes with and without the plug at 28.05 Hz is 1.625, which enables reliably distinguishing the two cases. It should be noted that during this laboratory test, the testing medium was fully elastic, so the measured differences correspond to the changes in structure and not the changes in stress.

### 3.2. Field Experiments

The prototype of the seismic beacon on the scale of 1:3 was constructed and used during the first field experiments. The device was produced by the company Parka Tech s.r.o. in 2022. The diameter of the steel cylinder, which forms the casing of the transmitter, was 80 cm. The rotor is realized by a couple of steel bodies in the shape of cylinders. The mass of these bodies is 30 kg each. This prototype was used during experiments in 2022. However, during these experiments, the rotor produced high-frequency acoustic noise, due to the moving of these cylinders across the small irregularities of the path. To avoid this noise, the moving bodies were exchanged with other ones of rotational ellipsoids shape and also the path was replaced with smoother one. This improved prototype was used during the experiments in 2023. The mass of ellipsoid bodies is 60 kg each. The bodies rotate around vertical axis with a frequency of 2.5 rev/s. That means that the basic frequency of transmitted seismic waves is 5 Hz, because during one revolution, there are two identical positions of the bodies. It is driven by an electric engine with very stable revolution controlled by a computer, which ensures the revolution with an accuracy 0.1%.

**Figure 4:**
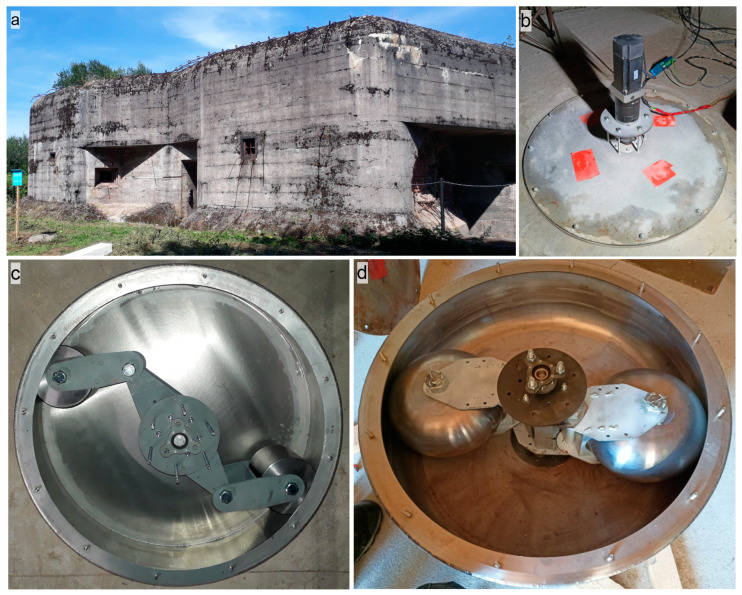
Installation of the seismic beacon prototype in the underground of the old military bunker. (**a**) Bunker from outside, (**b**) Seismic beacon during measurement, (**c**) open seismic beacon prototype, which was used in 2022, (**d**) prototype used in 2023.

**Figure 5:**
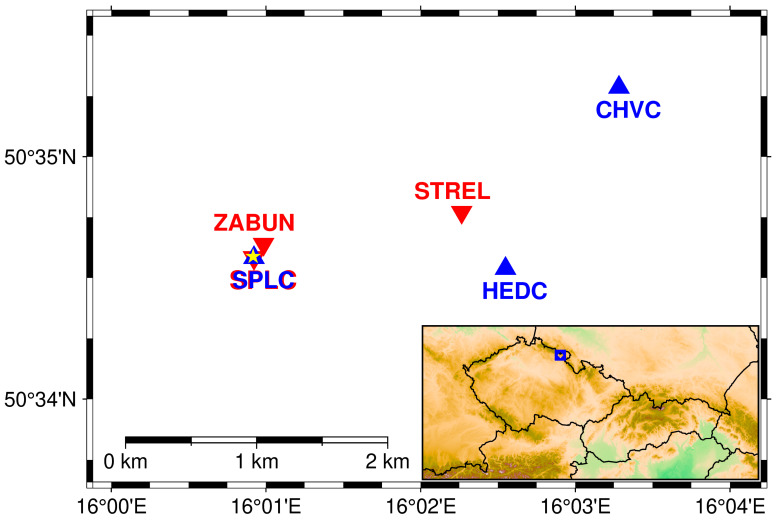
Seismic stations during measurements in 2022 (red triangles) and 2023 (blue triangles). The station SPLC was identical for both experiments. The yellow asterisk indicates the position of the generator of the seismic beacon. The area of the study is highlighted by a blue rectangle on the topography map of the Czech Republic and its surroundings on the bottom-right panel.

On 20 July 2022, the seismic beacon was installed to the old military concrete bunker in East Bohemia (50.5765° N, 16.0152° E). The bunker is located in the seismoactive area of the Hronov Poříčí Fault Zone (HPFZ) [28,29]. The seismic beacon was fixed in the floor of underground deck of the bunker, 4 m under the surface, Figure 4. Special type of concrete was used to make the contact between the device and the floor firm enough to withstand long-lasting vibrations of the device. After two weeks of concrete hardening, the first experiment took place on 4 August 2022 (Figure 5, red triangles).

**Figure 6:**
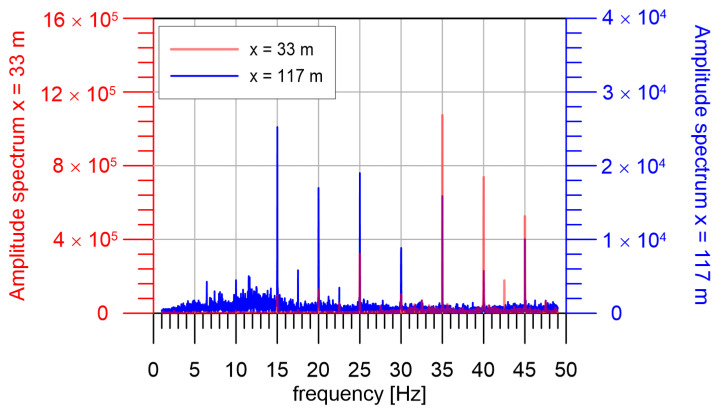
Spectra at two geophones at the distance 33 m and 117 m. The fundamental frequency of 5 Hz is very weak compared to the higher frequency harmonics in the area close to the source.

First, we investigated the attenuation of the seismic waves produced by the seismic beacon. We used a 24-channel seismic apparatus Geometrics Geode with 24 vertical 4.5 Hz geophones. The first geophone was at a distance of 25 m (azimuth 122°), and the last one at a distance of 117 m (azimuth 138°). The step between receivers was 4 m and the profile was situated fit between stations SPLC and ZABUN. The sampling frequency was 250 Hz and we analyzed 4.5 h intervals when the seismic beacon was in operation. The comparison of amplitude spectra at the third and the last geophone is in Figure 6. We can see that the seismic beacon generates a basic frequency of 5 Hz and very strong higher harmonics from 10 to 45 Hz. We can approximate the dependence of amplitude on distance and frequency by the formula:

(1) $$A(f,R)=A_{0}(f)\;e^{-\alpha \left(R-R_{0}\right)}$$

where *f* is a frequency, *R* is an epicentral distance, *R*_0_ is a reference distance (e.g., 100 m), *A*_0_*(f)* is an amplitude at reference distance, and *α* is an attenuation coefficient.

**Figure 7:**
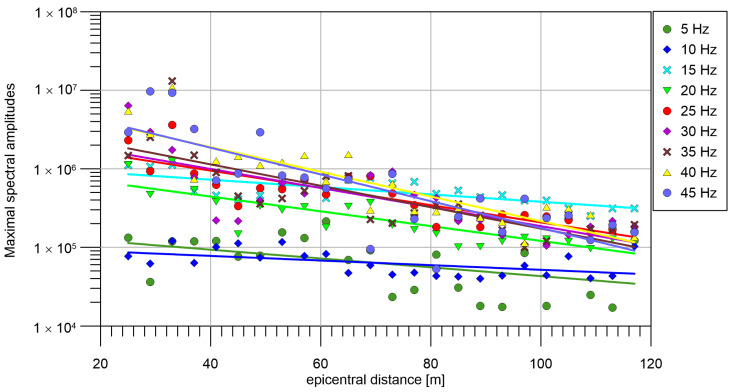
Maximum spectral amplitudes and their fit by exponential function.

**Figure 8:**
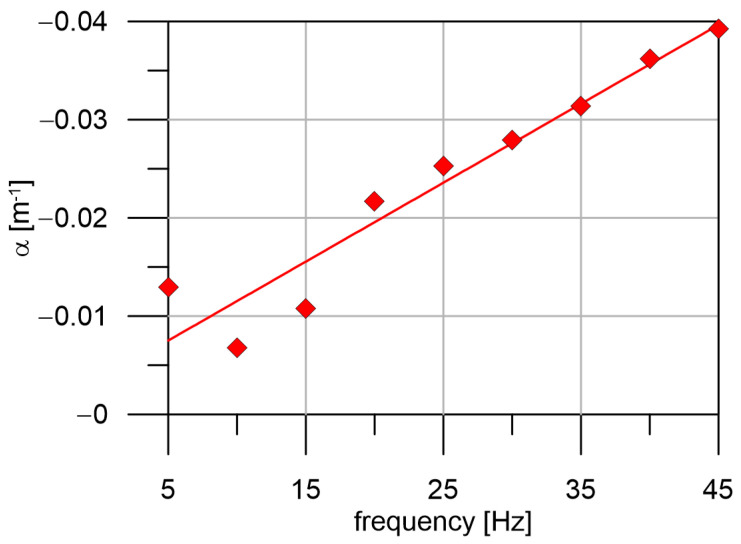
Frequency dependence of attenuation coefficient α.

The amplitudes at individual geophones (dependence on epicentral distance) are shown in Figure 7. The amplitudes vary significantly along the profile. We fitted it with an exponential function (1) (line in a logarithmic scale). The dependence of attenuation coefficient α, which was computed with help of Formula (1) is shown in Figure 8. In general, α increases linearly with the frequency. However, for frequencies 10 Hz and 15 Hz, the α is lower than expected from linear approximation. That means waves with frequency 10 Hz and 15 Hz propagates with low attenuation, which is probably caused by the thickness and S-wave velocity of uppermost layer, which forms surface waves with strong amplitude at these frequencies.

**Figure 9:**
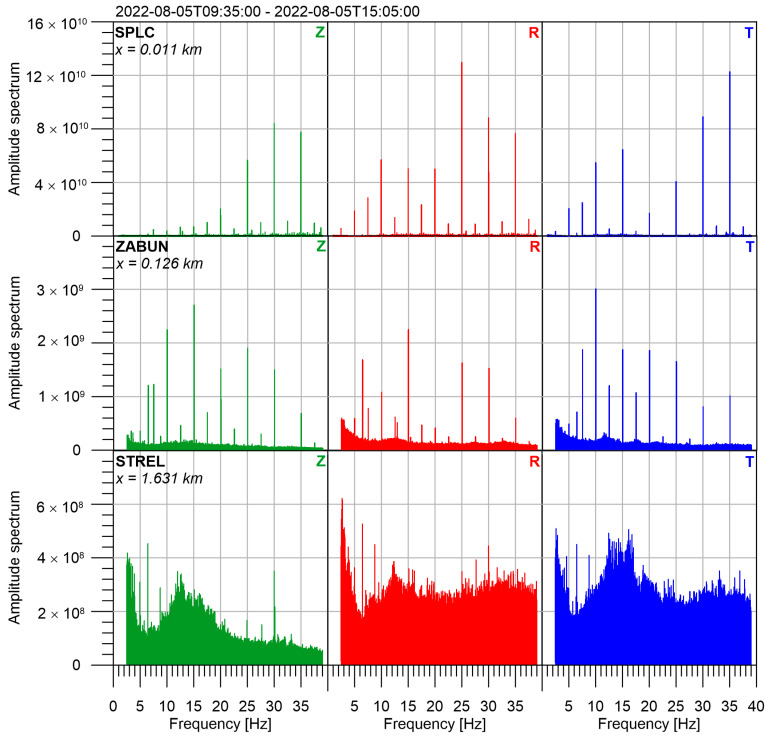
Spectra at three seismic stations SPLC, ZABUN, and STREL calculated from 5.5 h of measurement of the seismic beacon signal on 5 August 2022.

Based on Formula (1), we can predict the amplitudes at bigger epicentral distances. We realized that the amplitudes are comparable with seismic noise even at a distance of two kilometers. Therefore, we performed another experiment with three seismic sensors. We used broad-band seismometers Guralp CMG-40T. The sampling frequency was 250 Hz at all stations, the horizontal components were oriented to the north and to the east, and the vertical component had a plus sign upwards. The epicentral distances were 0.126 km (azimuth 139°) for station ZABUN, 1.631 km (azimuth 102°) for station STREL and the referenced sensor was in the distance 11 m (azimuth 50°) at the pillar of seismic station SPLC. The measurement was 5.5 h long. The data was filtered by a bandpass filter 1–40 Hz. The obtained amplitude spectra are in Figure 9. In the case of station STREL, the amplitudes of 5 Hz are visible at all 3 components, however, it is not much stronger than seismic noise. We consider the epicentral distance of STREL (1.6 km) as the maximum for this prototype of Seismic beacon. We computed the complex ratio (ratio of amplitudes and the difference of phases) between two distant stations and the reference one. These ratios are determined only by the velocity and attenuation between the seismic beacon and the stations. Therefore, it is sensitive to changes in the rock massif, change of stress, saturation by water, etc. Unfortunately, the construction of the prototype of the seismic beacon does not enable us to make long-lasting measurements. It was not robust enough and after several days, the bearings on the vertical axis became stuck and the seismic beacon has to be switched off. Nevertheless, these first tests proved that the signal from the model prototype used (which is on a scale 1:3) can be detected at a distance up to 1.6 km. The full-size seismic beacon will produce the seismic waves with energy much bigger. So, we expect the signal to be recognized at several tens of km distances.

The second field experiment took place on 26–27 June 2023 with an innovative Seismic beacon with ellipsoidal and heavier moving bodies and a smoother path. Three seismic stations were used as receivers during the experiment, Figure 5 (blue triangles). The first one (SPLC) was situated, as in year 2022, only 11 m from the transmitter in the same underground deck of the bunker, and served as a reference station. The second one (HED) was 1.928 km in the azimuth 87° in the short shaft of the old coal mine Hedvika, about 4 m from the portal. The third one (CHVC) was at a distance of 3.077 km in the azimuth 115°. It was placed on the same pillar as the permanent seismic station, which is operated in this locality in a small cellar about 3 m under the surface. All three seismic stations were identical with broadband sensor Trillium Compact TC120-SV1 and Earth Data EDR-210 digitizer. The sampling frequency was 250 Hz at all stations, the horizontal components were oriented to the north and to the east, and the vertical component had plus sign upwards. The gain was identical at all stations (1 digit is equal to the velocity of vibrations of 0.98 nm/s). The data was filtered by a bandpass filter of 1–40 Hz.

**Figure 10:**
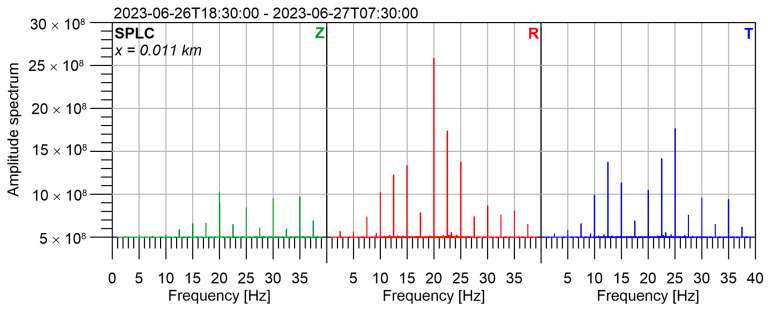
Amplitude spectrum at reference station SPLC, 10 m from the seismic beacon.

The experiment took place during the night to minimize the seismic noise. We performed measurements for 13 h (from 6:30 p.m. to 7:30 a.m.) at stations SPLC, HED a CHVC. The amplitude spectrum from the whole measurement at SPLC is in Figure 10.

**Figure 11:**
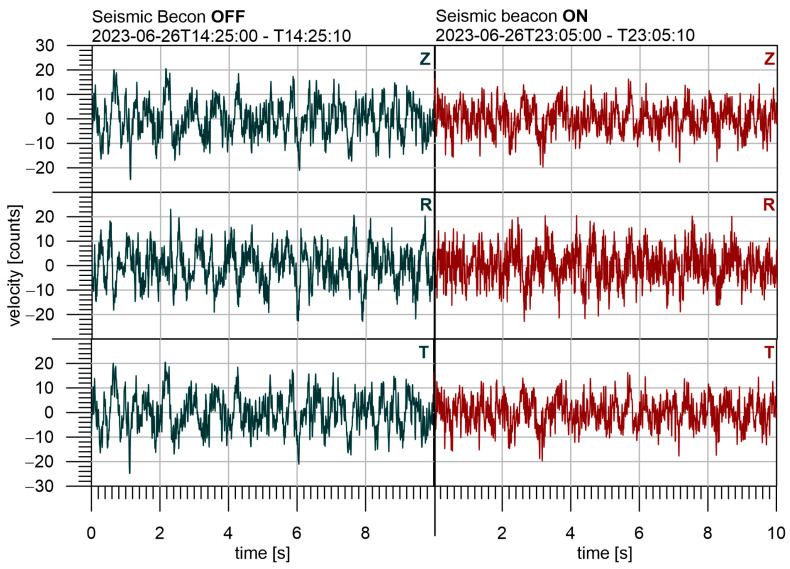
Comparison of seismograms at station HED with the seismic beacon off (left panel) and on (right panel). A low-pass filter at 40 Hz was applied.

We need to recognize the signal from the noise at the stations HED and CHVC. However, the power of the source is very low, and the transmitted seismic waves cannot be recognized in the seismograms directly. It is documented in Figure 11, where the short intervals of 10-s seismograms are compared at HED. The first seismogram is without the signal from the seismic beacon (it was off) and the second one is with the signal (it was on). It is important especially for other seismological observations, e.g., monitoring of weak local earthquakes, which should not be disturbed by seismic beacon operation. Earthquakes generate signals with a wide range of frequencies, while the seismic beacon generates waves of one fundamental frequency and its higher harmonics. In case the frequency ranges are similar, we can distinguish seismic beacon signal and apply a notch filter to detect such weak events. To minimize the disturbance of the desirable signal by the seismic beacon, it is crucial to set the fundamental frequency very carefully—ideally to avoid the frequency range of the presumed seismic signals.

**Figure 12:**
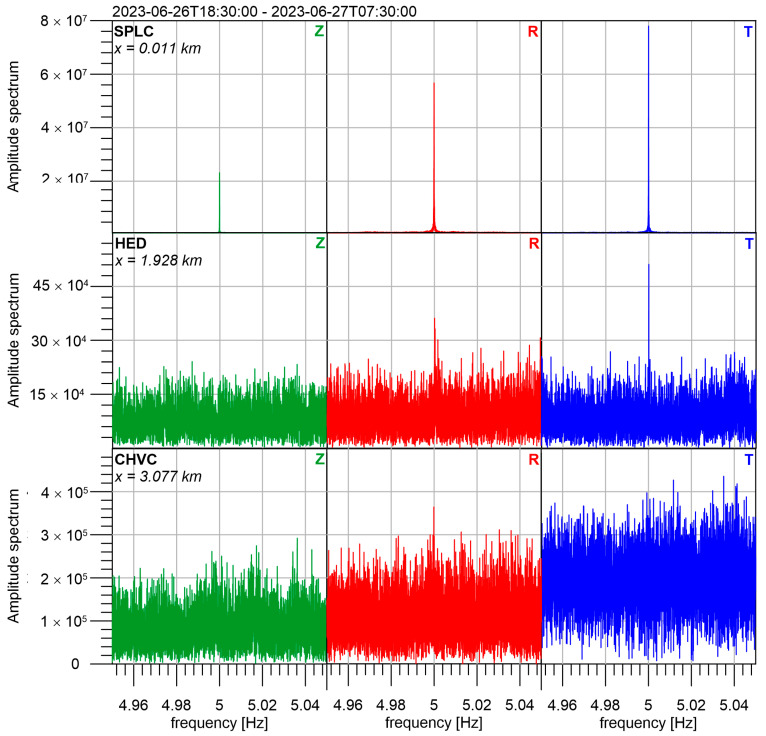
Amplitude spectra at 3 stations in the vicinity of the basic frequency 5 Hz.

**Figure 13:**
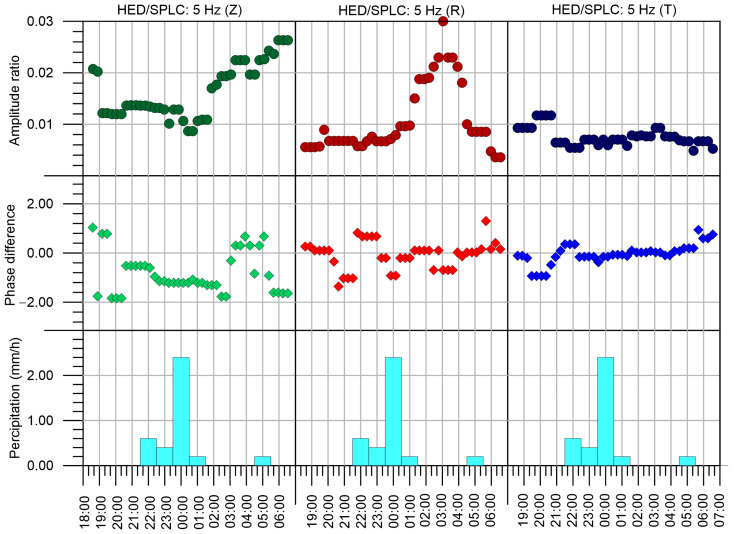
Amplitude ratio (upper panels) and phase difference (middle panels) at station HED for Z, R, and T components during 13 h measurement from 18:00 to 7:00. Moving window with a length 2.5 h is applied. The amount of precipitation at the close meteorological station in Velké Slavoňovice during measurement is shown in the bottom panels.

Amplitude spectra at the stations HED and CHVC around the investigated frequencies are depicted in Figure 12. We can see that the best signal-to-noise ratio is for 5 Hz at T component of HED. This depends on three factors. The first one is the amplitude of the transmitted signal at individual frequencies, the second one is the attenuation between the seismic beacon and the station, and the last one is the amplitude of seismic noise at the same frequency. As we would like to investigate mainly time changes of attenuation, we need to eliminate the influence of the other two factors. We used a special technique based on the analysis of a complex spectrum to reach this goal. We divided the whole measurement into 48 intervals to investigate the time changes. Each interval is 262,144 (2^18^) samples long, to enable effective application of the fast Fourier transform algorithm (FFT). It represents a time interval of 1048.6 s long. In each time interval, the ratio of complex spectra at stations HED/SPLC and CHVC/SPLC were computed. The results for investigated frequencies 5 Hz and 10 Hz are in Figure 13. The results were smoothed by the moving window technique with a length of 7 intervals (approximately 2 h). In each window median from 7 intervals is taken as a smoothed value. We can see that both amplitude ratios and phase differences have significant variations.

## 4. Discussion

The seismic beacon was designed to measure changes in a rock massif with long periods (years) or even permanent changes. For this goal, we need long-term measurement, which is planned for the next years. The tests in June 2023, described in this paper, lasted only 13 h, and their main purpose was to find optimal parameters of the transmitter and to prove that the signal from the seismic beacon of this size can be detected in seismic noise at the seismic stations in the distance of several km. We did not expect to find any variations in the rock massif. Surprisingly, variations both in amplitude ratio and phase difference in the measured data were found even during this one-night measurement. It was caused probably by the saturation of the soil layer with water, as heavy rain takes place in the locality for approximately two hours from 10 p.m. to 1 a.m. The precipitation data were obtained from the meteorological station Velké Slavoňovice “https://www.meteosvatonovice.cz/”(obtained on 29 July 2023), which is 6 km apart from the seismic beacon. It documents the high sensitivity of the device to changes in the uppermost layer. This phenomenon is probably linked to the effect of filling the pores in the soil with water and hence change in pore pressure. Rainfall decreases seismic velocity through changes in effective stress, which is described, e.g., by [30,31,32]. A more detailed study of this phenomenon will be carried out in future experiments when additional data from rainy days will be available. Short-term responses to rainfalls were also observed within the monitoring of temporal variations in the seismic wave travel times by vibration source ACROSS [33] as well as in the study [34], where a piezoelectric transducer was used as the ultrasonic wave source.

The construction of the used prototype of the seismic beacon, unfortunately, did not enable longer measurements, as some parts were deformed and damage of the whole device was expected if the device will be in operation longer. The more robust construction of the device is necessary before a longer experiment will be performed.

A very important task is to select the appropriate fundamental frequency of the seismic beacon generator. The fundamental frequency should be within the range of low seismic noise and out-of-range of events that are required to be monitored for other purposes. It should be considered that during both laboratory and field tests, we found that prototypes emit waves with dominant energy in higher frequencies harmonics.

Due to the limited budget of our project, both field and laboratory prototypes were simplified compared to patented solution, and some elements were missing (e.g., heater, thermometer, vacuum pump). Nevertheless, even these simple prototypes successfully verified the functionality of the Seismic beacon.

Comparing the seismic beacon to other devices used for seismic waves generation during seismic prospecting, there are several significant differences. As the purpose of the seismic beacon is to detect time changes in rock massif and not the structure of the massif itself, the most important property is the time stability of generated seismic waves. We need both the same frequency and amplitude of the waves. It is reached by the special mechanical construction, stable temperature, and low pressure inside beacon and controlling of the engine revolution by computer. As the measurement using the seismic beacon has a long-lasted character, we do not need mobility of the device, which is needed, e.g., in the case of truck-mounted vibrators, e.g., [7,34,35,36,37].

As already stated in the introduction, the seismic beacon generator is similar to a seismic source called ACROSS, e.g., [12,13,14,15,16,17]. Both seismic sources can be described as sinusoidal vibrators. They are suitable mainly for long-term monitoring because sinusoidal vibration is less destructive for surrounding ground than impulses. ACROSS source is designed to generate sinusoidal waves by a rotating eccentric mass around an axis. The sinusoidal wave is generated by the centrifugal force of the source, which is firmly fixed to the ground [12]. The majority of published measurements with system ACROSS use frequencies from 10 Hz to 50 Hz. However, in the study [38] the ACROSS source was operated between 3.5 Hz and 7.5 Hz for 10-year observation in Japan and they detected travel time variation as a secular advance and a co-seismic steplike delay at the time of the 2011 Tohoku earthquake. On the opposite, the seismic beacon generator contains two rolling elements moving around a vertical axis along a circular path, and it is designed to generate especially low frequencies of 1–5 Hz.

## 5. Conclusions

The seismic beacon is a system consisting of a harmonic seismic-wave transmitter and seismographs as receivers. It was designed to measure changes in a rock massif, especially stress changes caused by earthquakes and magma movements during volcanic explosions, but it can detect also changes in soil water saturation and fluid movement in Earth’s crust.

Laboratory tests verified the seismic beacon’s functionality. The laboratory model enables to generate seismic waves under different conditions, which are modified by a plug that can be filled with various materials. The tests confirm that higher harmonics of basic frequency are observed and their amplitude can be very strong. Amplitudes of these higher harmonics strongly depend on the properties of the medium, where seismic waves propagate.

The first field tests with a prototype in scale 1:3 proved that the signal can be recognized in seismic noise at epicentral distances up to 3 km. It enables the detection of changes in amplitude ratio and phase difference between seismographs. It corresponds to changes in the seismic velocities and in attenuation between transmitter and seismic stations. We observed variations in amplitude ratio and phase difference that can be connected with the amount of precipitation. The exact mechanism of this observation requires additional field measurement and further analysis.

## 6. Patents

Seismic Beacon has Czech patent PV 2020-642, international application No. PCT/CZ2021/050140).

## Data Availability

The data presented in this study are available on request from the corresponding author.
